# Prothrombin complex concentrate in the reduction of blood loss during orthotopic liver transplantation: PROTON-trial

**DOI:** 10.1186/1471-2482-13-22

**Published:** 2013-07-01

**Authors:** Freeha Arshad, Brigitte Ickx, Rachel T van Beem, Wojciech Polak, Frank Grüne, Frederik Nevens, Minna Ilmakunnas, Anna-Maria Koivusalo, Helena Isoniemi, Paul FW Strengers, Henk Groen, Herman GD Hendriks, Ton Lisman, Jacques Pirenne, Robert J Porte

**Affiliations:** 1 Department of Hepatobiliary Surgery and Liver Transplantation, University Medical Center Groningen, University of Groningen, Groningen, The Netherlands; 2 Department of Anaesthesiology, Hospital Erasme Brussels, Free University of Brussels, Brussels, Belgium; 3 Sanquin Blood Supply Foundation, Amsterdam, The Netherlands; 4 Department of Transplantation Surgery, Erasmus Medical Center Rotterdam, Erasmus University Rotterdam, Rotterdam, The Netherlands; 5 Department of Anesthesiology, Erasmus Medical Center Rotterdam, Erasmus University Rotterdam, Rotterdam, The Netherlands; 6 Department of Hepatology, University Hospital Leuven, Catholic University Leuven, Leuven, Belgium; 7 Department of Anaesthesiology and Intensive Care, Hospital District of Helsinki and Uusimaa, Helsinki, Finland; 8 Department of Transplantation and Liver Surgery, Hospital District of Helsinki and Uusimaa, Helsinki, Finland; 9 Department of Epidemiology, University Medical Center Groningen, Groningen, The Netherlands; 10 Department of Anaesthesiology, University Medical Center Groningen, University of Groningen, Groningen, The Netherlands; 11 Department of Abdominal Transplant Surgery, University Hospital Leuven, Catholic University Leuven, Leuven, Belgium

**Keywords:** Orthotopic Liver Transplantation, Prothrombin Complex Concentrate, Haemostatis, Bleeding, Blood Loss, Transfusion Requirements, Cirrhosis

## Abstract

**Background:**

In patients with cirrhosis, the synthesis of coagulation factors can fall short, reflected by a prolonged prothrombin time. Although anticoagulants factors are decreased as well, blood loss during orthotopic liver transplantation can still be excessive. Blood loss during orthotopic liver transplantation is currently managed by transfusion of red blood cell concentrates, platelet concentrates, fresh frozen plasma, and fibrinogen concentrate. Transfusion of these products may paradoxically result in an increased bleeding tendency due to aggravated portal hypertension. The hemostatic effect of these products may therefore be overshadowed by bleeding complications due to volume overload.

In contrast to these transfusion products, prothrombin complex concentrate is a low-volume highly purified concentrate, containing the four vitamin K dependent coagulation factors. Previous studies have suggested that administration of prothrombin complex concentrate is an effective method to normalize a prolonged prothrombin time in patients with liver cirrhosis. We aim to investigate whether the pre-operative administration of prothrombin complex concentrate in patients undergoing liver transplantation for end-stage liver cirrhosis, is a safe and effective method to reduce perioperative blood loss and transfusion requirements.

**Methods/Design:**

This is a double blind, multicenter, placebo-controlled randomized trial.

Cirrhotic patients with a prolonged INR (≥1.5) undergoing liver transplantation will be randomized between placebo or prothrombin complex concentrate administration prior to surgery. Demographic, surgical and transfusion data will be recorded. The primary outcome of this study is RBC transfusion requirements.

**Discussion:**

Patients with advanced cirrhosis have reduced plasma levels of both pro- and anticoagulant coagulation proteins. Prothrombin complex concentrate is a low-volume plasma product that contains both procoagulant and anticoagulant proteins and transfusion will not affect the volume status prior to the surgical procedure. We hypothesize that administration of prothrombin complex concentrate will result in a reduction of perioperative blood loss and transfusion requirements. Theoretically, the administration of prothrombin complex concentrate may be associated with a higher risk of thromboembolic complications. Therefore, thromboembolic complications are an important secondary endpoint and the occurrence of this type of complication will be closely monitored during the study.

**Trial registration:**

The trial is registered at http://www.trialregister.nl with number NTR3174. This registry is accepted by the ICMJE.

## Background

The liver is the site of synthesis of a large part of the proteins involved in the hemostatic system. When the function of the liver is reduced due to acute or chronic liver disease, the hemostatic system can be heavily affected. In patients with cirrhosis, both procoagulant and anticoagulant hemostatic changes have been described, leading to a new rebalanced state [1]. First of all, in the primary hemostasis, platelet number and function can be significantly affected, mostly due to impaired production of thrombopoietin by the liver, reduced platelet survival and increased in platelet consumption [2-4]. The defects in platelet function however, can be compensated by the elevated levels of Von Willebrand factor (VWF), an important endothelial-derived platelet adhesion protein [5,6]. Secondly, there is a decrease in coagulation factors synthesized by the liver. In particular the levels of vitamin K dependent coagulation factors II, VII, IX and X correlate negatively with the severity of disease [7]. However, not only levels of pro-coagulant proteins are decreased in liver disease, the liver also synthesizes coagulation inhibitors and both pro- and anti-fibrinolytic proteins, which are also affected. E.g., plasma levels of vitamin K dependent anti coagulation proteins C and S are decreased [8]. Additionally, in chronic liver disease, a hyperfibrinolytic status has been described [9], although not all studies agree [10]. This hyperfibrinolytic status may be due to decreased plasma levels of antiplasmin and thrombin-activatable fibrinolysis inhibitor, and to a dysbalance in tissue-type plasminogen activator and its inhibitor plasminogen activator inhibitor type 1 [11]. Furthermore, laboratory features of fibrinolysis include increased levels of markers of fibrinolytic activity such as D-dimers, but it must be noted that increased levels of these products may also be caused by accumulation as a result of decreased clearance [10].

Although the defects in coagulation factors would suggest that there is a bleeding tendency, both thrombotic events as well as bleeding complications may occur in patients with advanced liver disease. This might be explained by the fact that, although there is a rebalanced state, both procoagulant and anticoagulant proteins are decreased. The new rebalanced hemostasis is more precarious and susceptible for decompensation towards hypo- or hypercoagulability by factors such as infection, surgery, blood loss, transfusion, hypothermia etc. Furthermore, the bleeding tendency in chronic liver disease patients is much less predictable than in patients with a congenital defect in their coagulation system, e.g. hemophilia [1].

Laboratory tests in chronic liver disease, such as the prothrombin time (PT) and the international normalized ratio (INR), often suggest a hypocoagulable state. However, these tests do not represent the newly formed balance between pro- and anticoagulant proteins, since these tests are not sensitive for deficiencies of the anticoagulant proteins [12]. In contrast with the findings of these routine laboratory tests, the coagulation potential in cirrhotic patients appears to be normal or even superior to that of healthy individuals when tested by thrombin generation assays [13-15].

Orthotopic liver transplantation (OLT) is a major hemostatic challenge, which often requires transfusion of blood products. In the past decades blood loss during OLT has gradually decreased despite the fact that the conventional laboratory measurements like PT and INR still indicate hypocoagulability. It is unclear whether the prolonged PT and INR should be left out of consideration since studies have demonstrated that these measurements do not predict blood loss and transfusion requirements during OLT [16-18]. Furthermore thrombin generation assays during OLT, similar to thrombin generation assays in cirrhotic patients, indicate a normal to hypercoagulable status [19]. Nevertheless, excessive blood loss during OLT does still occur and transfusion of blood products during liver transplantation is associated with a higher risk of mortality, postoperative multi organ dysfunction, and reduced graft survival [20-22].

Clinical strategies to reduce blood loss include the use of blood products to correct pre-existing coagulopathy (assessed by prolonged PT and high INR) by transfusion of fresh frozen plasma (FFP), platelet concentrate, cryoprecipitate or fibrinogen, or antifibrinolytic agents to correct hyperfibrinolysis that may occur during the procedure. A major disadvantage of transfusion of blood products such as FFP is volume overload. To correct a prolonged PT several units of FFP are needed, which will result in an increase in central venous and portal (splanchnic) venous blood pressure, which may in fact increase the bleeding risk. Especially in patients with cirrhosis, who already have a relative fluid overload and increased splanchnic venous circulating volume, intravenous fluid administration will result in an increase in splanchnic venous pressure [23]. Portal and splanchnic hypertension are important risk factors for blood loss during OLT and several authors advocate not correcting pre-operative routine coagulation test by infusion of large volumes of FFP, maintaining a low venous pressure by fluid restriction, or even phlebotomy to prevent excessive blood loss during OLT [24-28].

Prothrombin complex concentrates (PCC) are hemostatically active highly purified concentrates, prepared from pooled plasma [29]. They contain four vitamin K-dependent clotting factors (F) II, VII, IX and X [30]. Previous studies have suggested that administration of PCC is an effective method to normalize a prolonged PT in patients with liver cirrhosis [31-33]. A major advantage of PCC is their low volume load. In contrast to FFP, administration of PCC is not associated with a fluid challenge and the subsequent risk of further increasing portal and systemic venous blood pressure. The question arises whether the administration of a low volume prohemostatic product such as PCC, prior to an invasive procedure in a patient with liver disease, is helpful in reducing blood loss.

PCCs may be related with an increased risk of thromboembolic complications, including venous thromboembolism, acute myocardial infarction, and disseminated intravascular coagulation [34,35]. The link between these reported thromboembolic events and PCC infusion has, however, often been brought into question [30]. Recently, PCC was also proposed for the management of massive peri- and postoperative bleeding [30,36-40]. In some of these reports additional administration of PCC seemed to reduce blood loss and mortality in patients suffering from massive bleeding, either treated with vitamin K antagonists or not. Furthermore, no thromboembolic events were reported. Composition of the currently and previously marketed PCC formulations differs and the thrombotic risk presumably depends on the composition of the product. The thrombotic risk of recently introduced formulations is incompletely evaluated. A review summarizing several aspects of PCC including safety concludes that thromboembolic events are rare and several recent case series have shown that high PCC doses (40 IU/kg) are safe even in high risk patients [41,42].

Cofact® (Sanquin Blood Supply Foundation, The Netherlands) contains balanced amounts of prothrombin complex factors, low levels of activated coagulation factors and a substantial amount of anticoagulant proteins C and S and antithrombin. Both products do not contain heparin. In 1973, Sanquin started with the large-scale production of its first generation of PCC. Since then, no thromboembolic events have been reported. In addition, the manufacturing of this plasma derived product contains 2 robust virus removal steps. The current indication for PCC is bleeding and perioperative prophylaxis of bleeding in acquired or congenital deficiency of (one of) the four vitamin K-dependent clotting factors. This includes the treatment of deficiencies of the four vitamin-K dependant-clotting factors in liver disease.

The ability of PCC to successfully reduce transfusion requirements in liver transplantation has not been established yet. The aim of this trial is to evaluate the hemostatic efficacy and safety of preoperative Cofact® administration (during induction of anaesthesia) on the initial requirement of hemostatic products during surgery in cirrhotic patients with severe coagulopathy undergoing a first liver transplantation.

## Methods/design

### Design and objectives

The study is designed as a multicenter, double blind, randomized placebo-controlled trial. The study is investigator-initiated.

The primary objective is to study the hemostatic efficacy of preoperative PCC administration in patients with cirrhosis and severe coagulopathy undergoing liver transplantation. The hemostatic efficacy will be primarily monitored by recording the need for RBC transfusion.

The secondary objective is to study the hemostatic safety of preoperative PCC administration in patients with cirrhosis undergoing liver transplantation. The hemostatic safety will be monitored by adverse event surveillance and laboratory measurements, with a special focus on thrombogenicity.

All study procedures during this study are summarized in Table 1.

**Table 1:** Timing of study procedures

**Parameters**				**Timepoints**													
	**SEH**	**Surgical procedure**					**Postoperative days**										
		**Anesthesia**	**30 minutes after intervention**	**Pre-anhepatic phase**	**Anhepatic phase**	**Reperfusion phase**	**0/1**	**2**	**3**	**4**	**5**	**6**	**7**	**11**	**14**	**30**	
12-leads ECG	x	According to local protocol							x				x			
Lab	x						x	x	x	x	x	x	x		x	x
Troponin	x						x									
Plasma sample	x	x	**x**	**1 hour after intervention**	**15 minutes after start phase**	**15 minutes after start phase**	x		x		x			x		
				**2 hours after intervention**		**end of surgery**										
Viral safety	x															
TEG/ROTEM					**15 minutes after start phase**	**30 minutes after start phase**										
Blinded INR			**x**				x		x				x			
Ultrasound hepatic vessels		According to local protocol									first 24 h after surgery					
Registration transfusion products			**x**	**x**	**x**	**x**			x				x			
Physical examination with focus on thrombotic and ischemic events																

### Investigational medicinal product (IMP)

The study drug is PCC (Cofact®, Sanquin, The Netherlands) and the placebo is NaCl 0.9%. The known and potential risks of PCC are described in the summary of product characteristics (SPC). In this population, the theoretical risk for thromboembolic complications is a special point of care.

### Endpoints

The primary endpoint will be the number of RBC units transfused during the OLT procedure and in the 24 hour post-surgery period, starting from arrival at the ICU. Other efficacy endpoints are (measured during surgery and during the 24-hour period post-surgery):

• The number of transfused units of fresh-frozen plasma

• The number of transfused units of platelet concentrate

• Fibrinogen concentrate administration

• Estimated blood loss

• Antifibrinolytic drugs

• Crystalloids or colloids administered

• Other escape medication used

• Estimated ascites loss

Safety is a secondary endpoint, safety parameters recorded are:

• All (Serious) Adverse Events with special focus on thromboembolic events

• General and liver-specific laboratory parameters

• Lowest pH, Ca++, lactate, temperature (during and within the first 24 h after surgery)

• Troponin levels, ECG and hepatic vessel ultrasound results

### Participating centers

Two Dutch medical universities are participating in this trial:

– University Medical Center Groningen (UMCG)

– Erasmus Medical Center Rotterdam (Erasmus MC Rotterdam)

Two Belgian medical universities are participating in this trial:

– University Hospitals Leuven (KULeuven)

– Hospital Erasmus Brussels

One Finish hospital is participating in this trial:

– Hospital District of Helsinki and Uusimaa (HUS Helsinki)

### Study subjects

Adult patients on the waiting list for liver transplantation because of cirrhotic liver disease and a prolonged INR (≥1.5) will be asked for informed consent. Inclusion and exclusion criteria are summarized in Table 2. Upon admission in the participating center for OLT, the inclusion and exclusion criteria and the informed consent will be double-checked. Patient with co-morbidities or a medical history that increases the risk for bleeding or thrombotic events during and after OLT will be excluded from participation. All patient characteristics, including demographic data, medical history and type of liver disease will be recorded. Participating patients will be monitored in a follow up of 30 days after inclusion.

**Table 2:** Inclusion and exclusion criteria

**Inclusion criteria**	**Exclusion criteria**
Age ≥18 years	Previous liver transplantation
Eligible for OLT	Split liver transplantation
INR ≥1.5	Heterotopic liver transplantation
Written informed consent	Scheduled multiorgan tranplantation
	Scheduled living-donor transplantation
	Renal insufficiency requiring dialysis
	Documented congenital coagulation disorders
	Documented history or presence of arterial or venous thrombosis
	Treatment with vitamin K antagonists
	TIPS (transjugular intrahepatic portosystemic shunt)
	Fulminant hepatic failure
	Documented coronary artery disease
	Documented thrombophilia

### Randomization and blinding

The surgical and anesthesiological team must remain blinded for the intervention during the entire duration of the trial. Randomization will be performed using an randomization website. We will stratify per center, and for patient age and gender. Randomization and preparation of the Investigational Medical Product (IMP), whether this is PCC or placebo, will be performed by an independent employee who is not involved in this trial, in the OLT or in post-operative care. The IMP will be delivered in a blinded syringe to the anaesthesiologist and administered by the anaesthesiologist.

### Intervention and dosage

The IMP will be administered during induction of anaesthesia, maximum 30 minutes prior to incision. The IMP, whether this is PCC or placebo will be administered according to the SPC of Cofact®/PPSB-D, intravenously and with a recommended infusion speed of 2 ml per minute. The dosage will be determined according to bodyweight, preoperative INR and target INR (≤1.5), as recommended in the SPC of Cofact® and as illustrated by Table 3. The same calculated dosage applies for the placebo.

**Table 3:** Dosing of PCC according to INR and bodyweight to reach an INR < 1.5

**Body**	**Initial INR**												
**weight**	**4.8**	**4.2**	**3.6**	**3.3**	**3.0**	**2.8**	**2.6**	**2.5**	**2.3**	**2.2**	**2.0**	**1.8**	**>1.5**
50 kg	60	50	50	50	40	40	30	30	30	30	30	20	20
60 kg	70	60	60	60	50	50	40	40	40	30	30	20	20
70 kg	80	70	70	70	60	60	50	40	40	40	30	30	20
80 kg	90	90	90	80	80	70	60	50	50	40	40	30	20
90 kg	100	90	90	90	80	80	70	60	50	40	40	30	20
100 kg	100	100	100	90	90	80	70	70	60	50	50	30	20

The dosages are calculated based on the factor IX concentration in PCC (the above dosage refers to ml of PCC). Calculated amounts are rounded mathematically on multiples of 10 ml and an upper limit of 60 or 100 ml in total was set (see tables above). The target INR values are recommended by the Federation of Dutch Thrombosis Services and are of the same order as English and German recommendations.

### Transfusion guidelines

The following guidelines have been established for homogenous transfusion policy in all participating centers:

• Fresh frozen plasma: During surgery the use of fresh frozen plasma is allowed in

• case of ongoing bleeding and hypocoagulability indicated by TEG or ROTEM as evaluated by the responsible anesthesiologist. Type of FFP, number of units and volume will be recorded in the case record form (CRF).

• RBC transfusions when hematocrit <25%: Adjustment can be performed at several time points; the units given before surgery do not count for the endpoint calculations, only those given during and directly after surgery (within 24 h of arrival at ICU) count for determination of endpoint. Number of units and volume of transfusion will be recorded in the CRF.

• Phlebotomy when hematocrit >30%: If the hematocrit post-operatively remains

• persistently elevated above 30, a phlebotomy will be performed to lower the hematocrit if the patient is stable and does not have an active bleeding.

• Platelet concentrate: Administration is permitted when platelet counts fall below 50,000/mm^3^ and in the presence o^f^ ongoing blood loss or if it is found necessary e.g. to prevent bleeding during OLT. Type, units and volume of platelet concentrate will be recorded in the CRF.

• Fibrinogen concentrate: Administration is permitted when fibrinogen levels are < 1.0

• g/L in the presence of ongoing blood loss. = > Transfuse 30 mg/kg.

• Tranexamic acid: During the procedure TEG of ROTEM will be used to evaluate fibrinolytic potential during the procedure (see chapter 7.1.3). Upon detection of hyperfibrinolysis on the TEG or ROTEM device in presence of ongoing bleeding due to presumed coagulopathy, a bolus of 1 gram tranexamic acid will be given. The bolus can be repeated in case of insufficient effect on TEG/ROTEM and clinical picture.

• Other transfusion products will be given according to the standard procedures at the individual liver transplantation centers.

• Topical hemostatic agents: Whenever clinically indicated topical administration of hemostatic drugs/agents (e.g. collagen) is allowed (recorded as concomitant medication).

• Cell savers: During the course of this trial, no cell savers will be used by participating patients.

• Escape medication: In case of a critical bleeding (by clinical judgment) during the liver transplantation procedure, treatment can be initiated in accordance with hospital guidelines and procedures e.g. antifibrinolytic drugs at habitual doses. All data on critical bleeding episodes should be reported as a serious adverse event. Type and amount of the escape medication and the justification will be recorded in the CRF.

• Enoxaparin or other low molecular weight heparin (LMWH): LMWH will be administered postoperatively as usual for deep venous thrombosis prophylaxis. Dosing will be according to routine local practice.

### Thromboelastography (TEG)

During the surgical procedure the transfusion will be guided by TEG of ROTEM (Rotational Elastometry). TEG/ROTEM has to be performed at least twice during the surgical procedure:

– 15 minutes after start anhepatic phase

– 30 minutes after start reperfusion phase

### Parameters during surgery

The following data will be recorded for participating patients:

– Donor characteristics

– Recipient characteristics

– Liver transplantation technique (conventional versus piggy-back)

– Use of veno-venous bypass

– Data on timing (cold-ischemia time, warm-ischemia time, duration anhepatic phase etc.)

– Surgical abnormalities and technical difficulties including heavy blood loss due to diffuse oozing or accidental vascular injury

– Ascites upon opening abdomen

– Lowest PH, Ca2+, lactate, temperature (during and within the first 24 hours of surgery)

– Transfusion requirements (during and within the first 24 hours of surgery)

– Medication (during and within the first 24 hours of surgery)

– Colloid/Crystalloid/Albumin/Gelatine infusion (during and within the first 24 hours of surgery)

### Laboratory parameters

– Prior to the OLT, an INR measurement will be performed to check if the patient still meets the inclusion criteria. After administration of the IMP, no routine INR measurements will be performed by the anaesthesiologist during the course of surgery, since measuring the INR will likely unblind the anaesthesiologist for the intervention.

– A blinded INR measurement will be performed (see section ‘Additional Study Procedures’) by an independent assistant 30 minutes after administration of the intervention and will only be available for an independent researcher. These measurements will be used during the interim analysis.

– Troponin levels are recorded just before and after surgery at ICU admission and the day after surgery, and in case of a suspected event due to clinical symptoms or ECG abnormalities during routine testing according to routine clinical practice.

– During OLT other laboratory and coagulation measurements (with the exception of the PT) may be performed according to local practice.

– Post-operatively, laboratory measurements presented in Table 4 will be performed from day 1–7 in the first week which is currently common practice in participating centers. From the second week, these laboratory measurements are only obligatory on day 14 and 30 (within a 24 hour timeframe).

**Table 4:** Laboratory parameters

**Hemostasis**	**Hematology**	**Clinical chemistry**	
Prothrombin Time	Hemoglobin (Hb)	C-reactive protein	Lactate dehydrogenase
Activated Partial Thromboplastin Time	Hematocrit	Glucose	Aspartate-aminotransferase
		Creatinine	Alanine-aminotransferase
Fibrinogen	Platelets	Urea	Alkaline Phosphatase
International Normalized Ratio	Leucocytes	Sodium	Albumin
		Potassium	Calcium
		gamma-glutamyltransferase	

### Surgical and anesthesiological procedure

There is no change in surgical or anesthesiological procedure from the current practise in participating centers.

### Postoperative procedure

The patient will be followed postoperatively according to local practise:

– Low molecular weight heparin as prophylaxis for deep venous thrombosis.

– Daily laboratory measurements in the first week

– Ultrasound of the hepatic vessels within 24 hours of surgery, at day 3 and day 7 postoperative

### Additional study procedures

The following additional procedures are included for the study subjects and are not part of the local practice:

• Prior to intervention and after intervention viral safety blood samples will be collected

• During the OLT-procedure, 30-minutes after administration of the IMP, a blinded INR measurement will be performed. The result of this measurement will be kept blinded for the anesthesiology and surgical team but will be recorded by an independent researcher. These results will be used during the interim analysis to investigate whether the INR in the PCC-group reached a value below 1.5 or whether the dosages of PCC need adjusting.

• Troponin levels are recorded just before and after surgery at ICU admission and the day after surgery.

• At 12 time points an additional blood sample will be collected from the patient for satellite studies on e.g. thrombin generation and fibrinolysis after completion of this trial. See Table 5 for the time points.

**Table 5:** Time point of additional blood sample collection

**Phase under study**	**Timing of blood sample collection**
Baseline	1. upon admission for OLT
Intervention	2. prior to intervention, after induction of anaesthesia
	3. 30 minutes after intervention
Pre-anhepatic phase	4. 1 hour post-intervention
	5. 2 hours post-intervention
Anhepatic phase	6. 15 minutes after start anhepatic phase
Reperfusion phase	7. 15 minutes after start reperfusion phase
	8. end of surgery
Follow-up	9-12. postoperative days 1, 3, 5, and 11

• On day 3 and 7 physical examination is performed with special attention to clinical venous thromboembolic events. In case of suspected deep venous thrombosis Doppler ultrasonography and on suspicion of pulmonary embolism a CT-angiography will be performed. If indeed a thrombotic complication occurs in the deep venous system or in the pulmonary arterial circulation, therapy will be initiated according to the local practice and with regards to the patient’s post-transplant status.

• A 12-lead electrocardiogram performed on the day of treatment prior to the intervention and product administration and 3 and 7 days post surgery and on clinical indication.

### Statistics

#### Sample size

Based on experience in the participating centers, it is assumed that cirrhotic patients undergoing OLT have a mean transfusion requirement of 8 U RBC (SD = 4 U). This data was derived from a retrospective data analysis of transfusion requirements during OLT in the UMCG over 2008–2011. The sample size of this trial was chosen to detect a reduction of 2 units of RBCs with approximate 80% power. The following statistical formula for sample size calculation was used to determine the sample size. N = 16***σ***^2^ / d^2^.

With an expected 10% withdrawal or loss of study subjects we concluded that 70 patients in each group are required to demonstrate a difference between placebo and PCC administration.

#### Interim analysis

One interim analysis will be performed by the trial statistician after 70 patients (50% of the sample size) have been included.

The Sponsor has chosen different boundaries for the different endpoints for the interim analysis, giving more significance to early unfavourable trends then to early beneficial trends. These boundaries are more used as guidelines rather than absolute rules.

For the efficacy endpoint, the O’Brien-Fleming sequential boundary will be used to correct for the instability of efficacy parameters at the beginning of the trial. For a total number of 2 planned statistical analyses and one planned interim analysis a Z-value of 3 with a P of 0.0027 will be used to determine statistical significance during the interim analysis. During the final analysis a Z-value of 2 and a P-value of 0.045 will be used to determine statistical significance.

For the safety endpoints, we have chosen to use the Pocock sequential boundary, dictating a Z-value of 2 and thus a P-value of 0.045 to determine statistical significance in harm between the two groups during the interim analysis as well as the final analysis.

Besides the efficacy and safety endpoint, during the interim analysis, sample size assumptions will be monitored based on the transfusion requirements in the placebo- group and if needed the sample size will be adjusted. Also, results from the blinded INR-measurements 30 minutes after intervention in de PCC-group will be used to determine whether the PCC-dose sufficiently lowers the INR in this patients group of whether the dose needs adjusting during the further conduct of this trial.

The trial may be stopped early due to one of the following situations:

– Unacceptable safety concerns: The analysis shows significant (serious) adverse events in the treatment group compared to the placebo group.

– The results on the endpoint between treatment-group and placebo group are inconclusive and it is expected that no significance will be reached.

– The interim analysis shows a clear benefit in the treatment group compared to the placebo group.

– In case new external information arises that convincingly answers the study question or raises serious safety issues.

#### Statistical analysis

Statistical analyses will be performed using the statistical software package SPSS 19.0 (SPSS Inc, Chicago, IL). Two-sided tests using the 5% critical level will be used throughout. The Mann–Whitney U test will be used to determine a significant difference in transfusion needs between the 2 groups. Intergroup categorical data will be compared using Chi-square or Fisher exact test. Intergroup continuous data will be analyzed with parametric or non-parametric analysis methods depending on the presence/absence of Gaussian distribution. If required, appropriate corrections for multiple comparisons will be applied. Independent risk factors for bleeding diathesis and transfusion during OLT will be identified using uni- and multivariate logistic regression analysis. All variables that reached a P < = 0.05 in the univariate analysis will be included in the multivariate linear regression analysis. P <0.05 will be considered statistically significant.

### Labelling and storage of IMP

The study medication (both PCC and Placebo (0.9% NaCl)) will be prepared and labelled as study medication for the PROTON trial conform GMP annex 13. The studymendication will be delivered and distributed freely by the Company to all participating centers.

#### Drug accountability

The local pharmacy in each participating centre is responsible for the correct storage and management of medication. All study medication (used, partially used and unused) will be noted on the provided Drug Accountability Form. The vials with remaining content will be disposed after usage, a signed record of all used study medication will be retained for inspection by the Monitor after completion of the trial. No study medication may be dispensed to any persons except to the subjects enrolled in the trial.

### Additional burden and risk associated with trial participation

Since PCC contains procoagulant proteins, there is a theoretical risk of thromboembolic complications associated with administration of this product.

PCC is manufactured from human plasma from living blood donors and thus the risk for viral transmittable disease cannot fully be excluded. The risk for transmittance is minimized by testing and screening the donor for past exposure to certain viruses. By testing donors for the presence of certain current viral infection, and by inactivation and elimination of certain viruses, the risk for transmission is further reduced. In this trial, to monitor viral safety of the PCC product, pre-treatment and post-treatment blood samples will be taken and stored until 1 year after the last patient is out of the trial.

There is a small risk for allergic or anaphylactic reaction to the study product PCC. In case of such an event, treatment with PCC will be ceased immediately. A severe anaphylactic reaction can lead to shock and can be life threatening. There is expertise in every participating centre to manage and treat such an event.

The following additional procedures will be performed in patients participating in the PROTON-trial:

– Prior to intervention, blood samples for viral safety check will be collected

– The intervention during induction of anesthesia consisting of receiving a intravenous dose of placebo or PCC.

– An extra plasma sample (together with routine lab) taken from the patient during 12 time points in the trial with regards to patient safety.

– A blinded INR measurement will be performed 30 minutes after intervention

– On day 3 and 7 Physical examination will be performed with special attention to clinical venous thromboembolic events.

– Extra electrocardiographic examination will be performed twice during the trial.

### Safety reporting

In accordance to section 10, subsection 1, of the WMO, the Investigator will inform the subjects and the reviewing accredited medical ethics committee (MEC) if anything occurs, on the basis of which it appears that the disadvantages of participation may be significantly greater than was foreseen in the research proposal. The trial will be suspended pending further review by the accredited MEC, except insofar as suspension would jeopardize the subjects health. The Investigator will take care that all subjects are kept informed.

#### DSMB

To ensure the safeguarding of the included patients and the expected additional burden with trial participation, a DSMB with independent experts has been installed. The advice(s) of the DSMB will be provided on a regular basis after receipt, review and analysis of the interim and final efficacy and safety data to the Sponsor and Investigators and to the MEC that approved the protocol.

#### Withdrawal of individual subjects

Subjects and Investigator can decide to terminate participation in this trial at any time for any reason, particular safety reasons, if they wish to do so without any consequences.

#### Premature termination of the trial

The trial will be terminated prematurely if we find a high rate of Serious Adverse Events in the PCC-group during the interim analysis, possibly liable to the study product.

#### (Serious- ) adverse events and suspected unexpected serious adverse reactions (SUSAR)

All adverse events (AE) will be recorded in the CRF.

Since orthotopic liver transplantation is a surgical procedure with significant morbidity and mortality, we will report serious adverse events (SAE) once every three months in line listing to all accredited MEC and the DSMB. SAE resulting in death and suspected unexpected serious adverse reactions (SUSAR) will be reported real time to the Sponsor. The Sponsor will notify all accredited MEC, the DSMB and the Competent Authorities (CA) if necessary.

#### Annual safety report

In addition to the expedited reporting of SUSARs, the Sponsor will submit, once a year throughout the clinical trial, a safety report to the accredited MREC, CA, Medicine Evaluation Board and CA of the concerned Member States.

### Ethical considerations

This trial will be conducted according to the principles of the Declaration of Helsinki and according to Good Clinical Practice (GCP) guidelines. The trial has been approved by the Medical Ethical Committee (MEC) of the University Medical Center Groningen (UMCG) for all Dutch participating centers. The trial has to be approved by the MEC of participating centers in other countries before those centers can start with the inclusions. Prior to randomization, written informed consent has to be obtained from all participants.

All participating centers have an insurance policy for patients participating in this trial in their centers, according to the legal requirements in their country and all centers are required to inform the participating patients about the insurance policy.

### Data and material collection

All trial data will be anonymous collected in the Case Record Form (CRF). Patient will be ascribed a unique study number and a unique randomization number. Name, address and date of birth will be stored separately from the trial data. Independent monitoring will be established to ensure that this trial is conducted, recorded and reported in accordance with the protocol and GCP. The Investigators, members of the Health Inspection and members of the Medical Ethical Committee have access to personal data. With informed consent research data will be stored during 15 years. Human material will be stored non-traceable for 15 years, when informed consent is obtained. We expect that the stored material will be very valuable for future research.

## Discussion

Patients with end-stage liver disease have an abnormal hemostatic system which is characterized by reduced plasma levels of both pro- and anticoagulant proteins. This results in a hemostatic balance that is more fragile than in healthy persons, a phenomenon that has been described as the ‘rebalanced hemostatic system’ [1]. This rebalanced, yet more fragile, hemostatic system is compatible with the observed higher incidence of bleeding as well as thromboembolic complications in patients with end-stage liver disease. An additional factor that plays a critical role in the increased bleeding risk in these patients is the presence of portal hypertension. Especially, when patients with cirrhosis require an abdominal surgery, the risk of bleeding during the procedure is related to the increased pressure in the portal vein and the venous splanchnic circulation. Fluid overload and sequestration of volume in the venous circulation may contribute to this component of the increased bleeding tendency in patients with cirrhosis when undergoing surgery. Based on this knowledge a restrictive fluid infusion and blood product transfusion policy has been propagated in these patients [25-28].

PCC is a low volume plasma product that contains high concentrations of both pro- and anticoagulant proteins. In contrast to the traditional infusion of FFP to stimulate coagulation in cirrhotic patients, PCC does not add to the intravascular volume and therefore may be, theoretically, more effective in reducing bleeding complications than FFP infusion. The efficacy and safety of PCC in patients with liver cirrhosis requiring major abdominal surgery, however, has never been demonstrated.

A potential side effect of PCC in patients with cirrhosis may be a higher risk of thromboembolic complications. Thromboembolic complications are not infrequent after OLT and when thrombosis occurs in one of the hepatic vessels, this may lead to graft loss. In addition, patients undergoing OLT are at increased risk of developing central venous thromboembolic complications such as intracardiac thrombosis or pulmonary embolism [43]. The PCC we are going to use in our study is commercially available under the product name Cofact®. The efficacy and safety of Cofac® has been evaluated in several clinical studies and so far there has been no indication of an increased risk of tromboembolic events in participating patients [42,44-46]. However, most of these trials have been conducted in patients using vitamin K antagonists. There has been no trial reporting the use of Cofact® in cirrhotic patients. With regards to the nature of the product, the disturbed hemostasis in cirrhotic patients and the hemostatic disturbances caused by the OLT, we decided that thromboembolic complications will be an important secondary and safety endpoint in this trial.

For our primary endpoint we have chosen the number of transfused units of RBC. Blood loss will be registered as well, but should be considered as less stable parameter as blood loss will be mixed with ongoing loss of ascites.

The dosage of PCC will be determined for each individual patient, based on preoperative bodyweight and INR. We will not try to subtract preexisting ascites from the measured bodyweight as this will be hard to quantify preoperatively. Moreover, it can be expected that a proportion of the PCC will be lost through diffusion into the ascites. The current dosages of Cofact® are based on calculations used for the correction of a prolonged INR in patients receiving vitamin K antagonist therapy. We do not know yet whether the same dosing scheme can be used for patients with a prolonged INR due to liver insufficiency. To confirm adequate dosing of PCC we will perform a interim analysis of the INR measurements in stored plasma sampled taken in each patient 30 minutes after the administration of the trial medication. These INR measurements will be performed by a central laboratory and data will be analyzed by an independent researcher who is not involved in conduction of the study. The blinded INR measurements will be used to assess whether the dosing is sufficient or needs adjusting. No INR measurements will be performed during OLT by the local teams.

During the conduction of this trial the surgical and anesthesiological team, treating physicians, and all investigators have to remain blinded for the intervention. This raised a few practical challenges which will be dealt with by a standardized procedure for preparation of the study medication. On-site training and instructions will be given to the local investigators and teams to ensure adequate compliance with these procedures. One of the challenges we have dealt with is that PCC has a light blue color when dissolved. This blue color is visible when the product is in a syringe, but not when flushed through an infusion line. To keep the local teams blinded for the medication group we have chosen to wrap the syringes in aluminum foil before presenting them to the anaesthesiologist.

Randomization and preparation of the study medication will be performed outside the operating theatre by an independent assistant who is not further involved in the patient’s care or in the conduct of this trial.

## Abbreviations

AE: Adverse Event; CRF: Case Record Form; DSMB: Data Safety Monitoring Board; GCP: Good Clinical Practice; IMP: Investigational Medicinal Product In this study the IMP is either Prothrombin Complex Concentrate (Cofact®/ PPSB-D) or placebo (NaCl 0.9%); INR: International Normalized Ratio; LMWH: Low Molecular Weight Heparin; OLT: Orthotopic Liver Transplantation; PCC: Prothrombin Complex Concentrate; PT: Prothrombin Time; MEC: Medical Ethics Committee; (S)AE: (Serious) Adverse Event; SPC: Summary of Product Characteristics; Sponsor: The Sponsor is the party that commissions the organization or performance of the research, for example a pharmaceutical Company, academic hospital, scientific organization or Investigator. A party that provides funding for a study but does not commission it is not regarded as the Sponsor, but referred to as a subsidizing party, in this case the Company; SUSAR: Suspected Unexpected Serious Adverse Reaction; WMO: Medical-Scientific Research Act (in Dutch: Wet Medisch-Wetenschappelijk Onderzoek).

## Competing interests

Sanquin has provided a grant of €100.000 to the Sponsor (UMCG) for the appointment of a fulltime PhD-student to coordinate this multicenter. Also, Sanquin has agreed to deliver the study medication to all participating centers free of cost. Sanquin has no input on the conducts of the trial, the trial procedures and interpretation of trial data, but may aid merely in trial coordination.

The authors declare that they have no competing interests.

## Authors’ contributions

BI had the first idea for this study. FA drafted the manuscript. RvB, TL and RJP co-authored in writing the manuscript. FA, RvB, TL, RJP, BI, FN, JP, AMK, MI, HI, WP, PS and FG participated in the study design during several meetings and email correspondence. FA and HG performed the sample size calculation. All authors edited read and approved the final manuscript.

## Authors’ information

Dr. F. Arshad: physician researcher University Medical Center Groningen (UMCG); coordinating researcher PROTON-trial.

Dr. R.T. van Beem: Clinical Research Specialist Sanquin Blood Supply Foundation.

Drs. W. Polak: surgeon Hepatobiliary surgery and Liver Transplantation Erasmus Medical Center (Erasmus MC); Co-investigator PROTON-trial.

Dr. F. Grune: chief department of anesthesiology Erasmus Medical Center (Erasmus MC); Site Principal Investigator PROTON-trial.

Prof. Dr. J. Pirenne: chief department of Abdominal Transplantation Surgery University Hospitals Leuven (KULeuven); local Investigator PROTON-trial.

Prof. Dr. F. Nevens, chief department of Hepatology University Hospitals Leuven (KULeuven); local Investigator PROTON-trial.

Dr. M. Ilmakunnas, anaesthesiologist Intensive Care Unit Hospital District of Helsinki and Uusimaa; local Investigator PROTON-trial.

Dr. A.M. Koivusalo, chief Intensive Care Unit Hospital District of Helsinki and Uusimaa (HUS Helsinki); local Investigator PROTON-trial.

Prof. Dr. H. Isoniemi, head department of Transplantation and Liver Surgery Hospital District of Helsinki and Uusima (HUS Helsinki); Site Principal Investigator PROTON-trial.

P.F.W. Strengers: Medical Director at Sanquin Blood Supply Foundation

Dr. H. Groen, epidemiologist University Medical Center Groningen (UMCG.

Dr. H.G.D. Hendriks, anaesthesiologist University Medical Center Groningen (UMCG).

Prof. Dr. T. Lisman, head Section of Liver and Hemostasis, Surgical Research Laboratory, department of Hepatobiliary Surgery and Liver Transplantation at University Medical Center Groningen (UMCG); Co-investigator PROTON-trial.

Prof. Dr. B. Ickx: Anesthesiologist Erasmus Hospital Brussels; Principal Investigator PROTON-trial.

Prof. Dr. R.J. Porte: chief department of Hepatobiliary Surgery and Liver Transplantation at University Medical Center Groningen (UMCG); Principal Investigator PROTON-trial.

## Pre-publication history

The pre-publication history for this paper can be accessed here:

http://www.biomedcentral.com/1471-2482/13/22/prepub
